# Myocardial Deformation in Cardiac Amyloid Light-chain Amyloidosis: Assessed with 3T Cardiovascular Magnetic Resonance Feature Tracking

**DOI:** 10.1038/s41598-017-03699-5

**Published:** 2017-06-19

**Authors:** Rui Li, Zhi-gang Yang, Hua-yan Xu, Ke Shi, Xi Liu, Kai-yue Diao, Ying-kun Guo

**Affiliations:** 1 Department of Radiology, West China Hospital, Sichuan University, 37# Guo Xue Xiang, Chengdu, Sichuan 610041 China; 2 Department of Radiology, West China Second University Hospital, Sichuan University, 20# Section 3 South Renmin Road, Chengdu, Sichuan 610041 China; 3 Department of Radiology, Affiliated Hospital of North Sichuan Medical College, 63# Wenhua Road, Shunqing District, Nanchong, Sichuan 637000 China

## Abstract

Clinically, assessment of myocardial function is essential in patients with amyloid light-chain cardiac amyloidosis (AL-CA) to predict outcome and determine therapeutic approach. The aim of this study was to investigate the feasibility of cardiovascular magnetic resonance (CMR)-derived feature tracking algorithm for assessing left ventricular (LV) myocardial deformation in AL-CA, and to determine if these abnormal myocardial deformation parameters are correlated to impaired LV myocardial microvascular dysfunction. A total of 42 AL-CA patients, including 26 with preserved systolic function and 16 with impaired LVEF, and 35 healthy controls were enrolled and underwent CMR examination. Our result indicated that AL-CA patients had significantly reduced global peak strain (PS) (longitudinal, circumferential, and radial) (all P < 0.05). AL-CA patients with normal LVEF showed preserved longitudinal PS at apical and significantly reduced longitudinal PS at mid and basal segments. By Spearman’s rank correlation analysis, the LV regional radial, circumferential, and longitudinal myocardial deformation values were correlated to myocardial upslope and MaxSI in CA, regardless of LVEF. This study indicated that the abnormal LV myocardial deformation of AL-CA patients can be monitored using feature tracking CMR, even in those with preserved LVEF; and the myocardial deformation was associated with coronary microvascular dysfunction.

## Introduction

Cardiac amyloidosis (CA), characterized by extracellular deposition of pathologically insoluble proteins in heart tissue, is seen in more than one-half of the patients with systemic light-chain (AL) amyloidosis, and the major cause of death in these patients^1, 2^. Assessment of myocardial function is essential in patients with amyloidosis, as the extent and severity of cardiac involvement is the most significant indicator to determine patient prognosis^2–4^.

Clinically, the left ventricular ejection fraction (LVEF), reflecting global LV systolic function, has remained the reference standard for the evaluation of ventricular function. Reduced LVEF has been considered an independent predictor of cardiac mortality in AL amyloidosis^2^. However, subclinical dysfunction of the left ventricle can be observed by speckle tracking echocardiography in CA patients with preserved LVEF^5, 6^. Thus, LVEF is limited by its inability to assess regional function and less sensitive for the evaluation of early contractile impairment^7, 8^.

Cardiovascular magnetic resonance (CMR) tagging-derived myocardial strain values, which could measure cardiac muscle motion and deformation, have emerged as more sensitive indicators than EF for measurement of systolic function^9^. However, this established method is limited by time consuming and requiring additional sequence acquisition. As cine CMR imaging has relative high signal to noise, contrast to noise ratios and ability of unrestricted access to large fields of view, it could provide more accurate and reproducible assessment of changing in cardiac morphology and function^10^. The noninvasive evaluation of myocardial deformation imaging thus has been moved to post-processing of CMR cine in the clinical practice^11^. More recently, the CMR feature tracking imaging (FT-CMR), a new technology based on each tissue voxel motion tracking within the whole myocardium of routine steady state free precession (SSFP) cine images, showing a good correlation with CMR tagging and consuming much less scan time, could quantitatively detect global and segmental myocardium function^7, 12, 13^. However, to the best of our knowledge, few studies have evaluated myocardial dysfunction by CMR feature tracking in patients with AL-CA.

As previous studies have shown that myocardial strain imaging can identify systolic dysfunction before the LVEF is reduced^14–16^, we can presume that the CMR-derived feature tracking may be used to monitor the impairment of deformation in CA patients with normal LVEF. Moreover, disturbed microvascular function, observed as highly prevalent in patients with cardiac amyloisosis^17^, may play a role in LV function impairment. CMR imaging allows the simultaneous assessment of both perfusion and function by using one single study. Therefore, this study aimed to quantify the global and regional myocardial deformations by CMR feature tracking in AL-CA patients and to determine whether these deformation parameters are correlated to coronary microvascular dysfunction.

## Result

### Baseline characteristics

The baseline characteristics of the AL-CA patients and normal subjects are presented in Table 1. Of the 42 cardiac patients, 16 (38.1%) presented with impaired systolic function (LVEF < 50%). Patients with CA and impaired LVEF had significantly higher LV end diastolic volume (LVESV) and lower stroke volume (SV), and markedly increased LV mass than that of the normal subjects and the AL-CA patients with preserved LVEF (P < 0.05). Late Gadolinium Enhancement (LGE) was present in all CA patients, including the pattern of subendocardial (n = 14), transmural (n = 15), diffuse heterogeneous (n = 3), and patchy (n = 10). In patients with normal LVEF, the LGE distributed more in subendocardial area while the transmural LGE was mostly observed in those associated with reduced LVEF.

**Table 1:** Baseline Differences Between Cardiac Normal healthy, AL-CA patients with preserved and impairment LVEF.

	Normal subjects (n = 35)	CA with normal LVEF (n = 26)	CA with impaired LVEF (n = 16)
Age (Y)	50.83 ± 8.67	58.19 ± 8.56*	61.94 ± 11.92*
Male	17 (49)	14 (54)	8 (50)
**Echocardiography**			
Septal thickness (mm)	9.08 ± 1.60	15.27 ± 2.81*	17.94 ± 3.40*
E/A	1.3 ± 0.3	1.7 ± 0.7*	2.4 ± 0.8*^§^
E/E′	9.15 ± 1.92	16.79 ± 4.72*	21.46 ± 5.43*^§^
**CMR**			
LVEDV (ml)	132.94 ± 25.78	111.23 ± 25.98*	122.53 ± 23.56
LVESV (ml)	51.11 ± 16.76	46.71 ± 10.47	75.29 ± 17.64*^§^
LV SV (ml)	81.83 ± 15.52	64.52 ± 21.51*	47.24 ± 9.79*^§^
LVEF (%)	62.00 ± 6.8	57.42 ± 6.9	38.76 ± 5.7*^§^
LV mass (g)	87.07 ± 19.27	127.83 ± 31.51*	169.71 ± 47.34*^§^
**Presence of main LGE pattern**			
Subendocardial	—	10	4
Pathy		9	1
Diffuse	—	2	1
Transmural	—	5	10

Notes: The values are the mean ± SD, Numbers in the brackets are percentages. ^*^ *P* < 0.05 versus normal group; ^§^ *P* < 0.05 versus CA with preserved LVEF. AL-CA = light-chain amyloid cardiac amyloidosis; LV = left ventricular; EDV = end diastolic volume; ESV = end systolic volume; EF = ejection fraction; SV = stroke volume; LGE = Late gadolinium enhancement.

### Inter- and intraobserver agreement and variability of LV deformation parameter measurements

Inter- and intra-observer agreements for feature tracking analysis are shown in Table 2. For global measurement, feature tracking allowed for reproducible quantification of radial, circumferential, and longitudinal peak systolic strains (PS), showing intra- and inter-observer coefficient of variation (CV) of 7.75% and 11.77% for radial strain, −2.32% and −8.28% for circumferential strain, and −2.75% and −8.48% for longitudinal strain. On slice level, the variations increased to 6.91% and 14.75% for radial strain, −4.42% and −17.22% for circumferential strain, and −7.57% and −18.24% for longitudinal strain. The regional reproducibility was best at the basal and mid-ventricular levels and worst at the apex. Compared with global analysis, the regional strain values had lower intra- and inter-observer reproducibility in longitudinal (*P* = 0.028 and *P* = 0.007, respectively) and circumferential direction (*P* = 0.03 and *P* = 0.013, respectively).

**Table 2:** Intra-observer and Inter-observer coefficient of variation of CMR Feature Tracking.

	Global		Basal		Mid-ventricular		Apical	
	Intra-	Inter-	Intra-	Inter-	Intra-	Inter-	Intra-	Inter-
**Longitudinal**								
PS (%)	−2.75	−8.48	−7.57	−9.39	−9.05	−18.24	−11.48	−17.90
PSSR (1/s)	−5.84	−12.41	−8.69	−12.11	−10.54	−16.61	−10.94	−22.91
PDSR (1/s)	5.04	11.20	11.09	15.93	11.31	15.24	12.24	16.23
**Circumferential**								
PS (%)	−2.32	−8.28	−4.42	−9.29	−5.84	−13.82	−4.38	−17.22
PSSR (1/s)	−3.95	−11.75	−7.54	−10.62	−10.66	−18.13	−5.16	−17.51
PDSR (1/s)	7.25	12.86	5.32	14.91	6.36	14.70	7.90	20.91
**Radial**								
PS (%)	7.75	11.77	6.91	12.45	9.75	11.70	8.08	14.75
PSSR (1/s)	8.45	17.04	13.25	19.54	14.36	22.07	11.57	20.48
PDSR (1/s)	−8.40	−15.48	−15.25	−15.13	−9.89	−14.18	−11.00	−13.41

Note: The coefficient of variation is given in %. PS = Peak Strain; PSSR = Peak Systolic Strain Rate; PDR = Peak Diastolic Strain Rate.

### Global and regional strain analysis in AL patients and normal controls

As is shown in Table 3, the global radial, circumferential, and longitudinal PS were significantly decreased in AL-CA patients compared with normal subjects (all *P* < 0.017). In addition, patients with AL-CA and reduced LVEF associated with lower radial, circumferential, and longitudinal peak systolic strain rate (PSSR). With regard to diastolic function, there were significant differences in all assessed peak diastolic strain rate (PDSR) (all *P* < 0.0001) between normal controls and CA patients regardless of LVEF. However, no significant differences of circumferential and longitudinal PDSR were observed between patients with and without decreased LVEF (*P* = 0.1 and *P* = 0.041, respectively).

**Table 3:** Values for left ventricular deformation parameters obtained using feature tracking for global values of all study groups.

	Normal controls (n = 35)	CA with normal LVEF (n = 26)	CA with impaired LVEF (n = 16)
**Longitudinal**			
PS (%)	−16.33 ± 2.33	−11.92 ± 2.98*	−7.13 ± 2.55*^§^
PSSR (1/s)	−0.83 ± 0.16	−0.83 ± 0.23	−0.54 ± 0.24*^§^
PDSR (1/s)	1.13 ± 0.24	0.65 ± 0.42*	0.55 ± 0.20*
**Circumferential**			
PS (%)	−17.84 ± 2.00	−13.13 ± 2.64*	−8.24 ± 2.90*^§^
PSSR (1/s)	−0.96 ± 0.18	−0.89 ± 0.25	−0.62 ± 0.23*^§^
PDSR (1/s)	1.25 ± 0.25	0.76 ± 0.47*	0.68 ± 0.26*
**Radial**			
PS (%)	42.79 ± 10.29	34.98 ± 7.44*	21.66 ± 6.43*^§^
PSSR (1/s)	2.56 ± 0.72	1.97 ± 0.97*	0.86 ± 0.55*^§^
PDSR (1/s)	−3.33 ± 1.14	−1.39 ± 1.27*	−0.97 ± 0.47*^§^

Notes: The values are the mean ± SD, other abbreviations are the same as in Tables 1 and 2. ^*^ *P* < 0.017 versus normal group; ^§^ *P* < 0.017 versus CA with normal LVEF.

**Figure 1:**
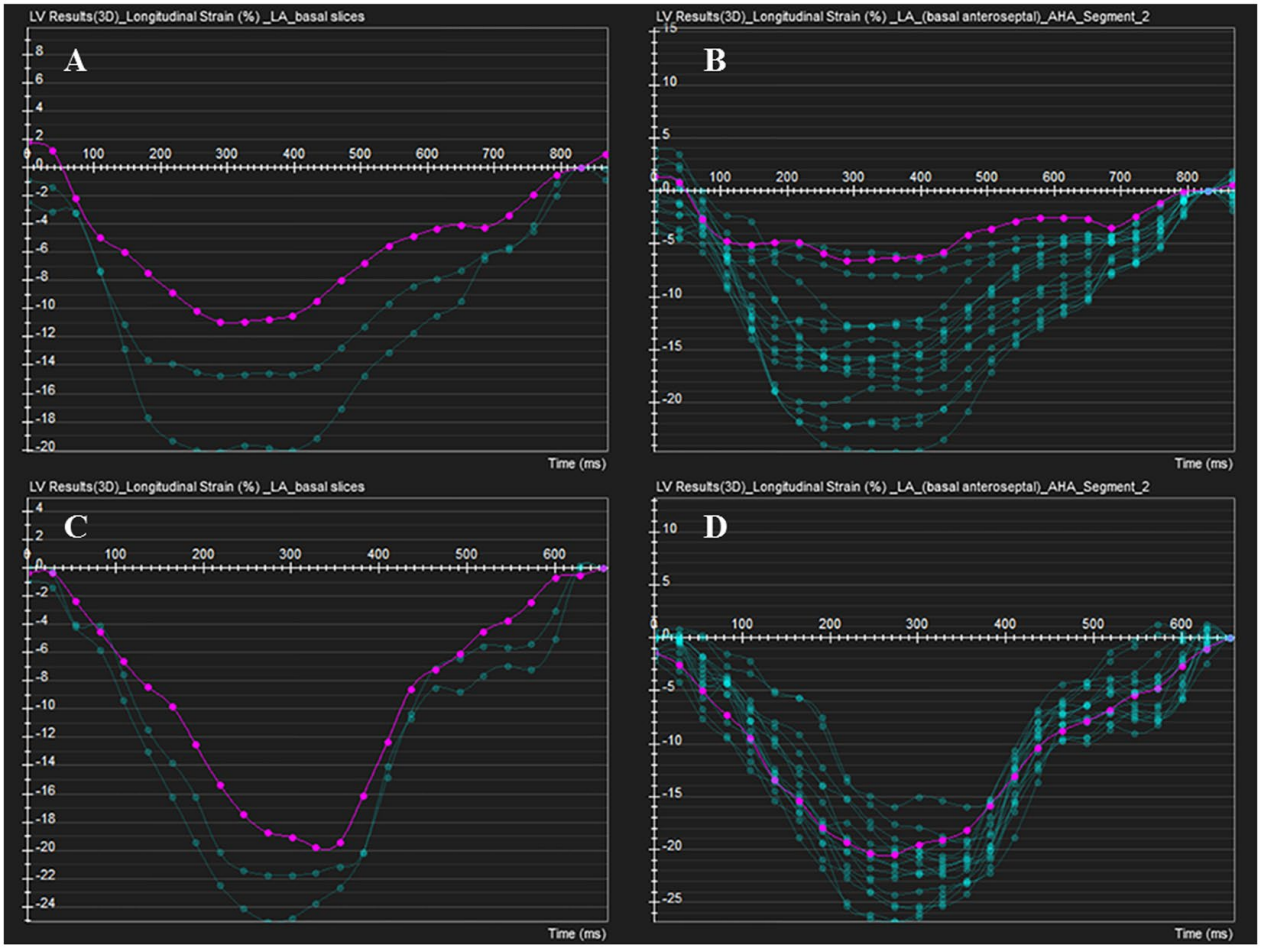
Feature tracking variables of longitudinal strain in AL-CA with preserved LVEF and normal control. (**A**,**B**) Red curve shows reduced longitudinal PS in LV base and basal anteroseptal segment, while the preserved longitudinal PS in apical segment could be observed. (**C**,**D**) No reduction of longitudinal PS in any three slices in normal control.

The regional deformation parameters among normal controls and patients with cardiac amyloidosis are provided in Table 4. On segmental levels, CA patients with preserved LVEF had significantly lower longitudinal and circumferential PS in basal and mid-ventricular slices (both *P* < 0.0001), and reduced radial PS in the basal slice (*P* < 0.0001) compared with normal subjects. The apical longitudinal PS, however, was decreased in CA patients with normal LVEF, but did not reach statistical significance compared with normal controls (*P* = 0.069), which was suggestive of apical sparing of longitudinal strain (Fig. 1). CA patients who progress to impaired LVEF are often accompanied with reduced apical longitudinal and circumferential PS in this study. There was also a significant difference in longitudinal PS from the base to apex gradient in patients with CA (All *P* < 0.01). However, this baso-apical strain gradient was not observed in circumferential or radial directions. In addition, the reduced diastolic function in CA patients regardless of LVEF could be observed, reflecting by relatively lower longitudinal, circumferential, and radial PDSR in basal, mid-ventricular, and apical segments (all *P* < 0.01).

**Table 4:** Values for left ventricular deformation parameters obtained using feature tracking for slice values of all study groups (basal, mid, apical).

	Normal controls (n = 35)	CA with normal LVEF (n = 26)	CA with impaired LVEF (n = 16)	Normal controls (n = 35)	CA with normal LVEF (n = 26)	CA with impaired LVEF (n = 16)	Normal controls (n = 35)	CA with normal LVEF (n = 26)	CA with impaired LVEF (n = 16)
	Basal			Mid-ventricular			apical		
**Longitudinal**									
PS	−13.72 ± 3.45	−7.71 ± 3.08*	−5.66 ± 2.42*^§^	−19.52 ± 2.83	−10.37 ± 3.62*	−7.08 ± 4.00*^§^	−17.43 ± 2.93	−16.07 ± 3.70	−9.82 ± 3.04*^§^
PSSR	−0.86 ± 0.32	−0.82 ± 0.24	−0.46 ± 0.15*^§^	−1.04 ± 0.22	−0.92 ± 0.37	−0.64 ± 0.37*	−1.14 ± 0.32	−1.14 ± 0.38	−0.85 ± 0.57*^§^
PDSR	0.98 ± 0.33	0.69 ± 0.24*	0.42 ± 0.34*^§^	1.48 ± 0.35	0.74 ± 0.44*	0.58 ± 0.30*	1.42 ± 0.46	1.12 ± 0.35*	0.83 ± 0.33*^§^
**Circumferential**									
PS	−17.12 ± 2.15	−12.22 ± 2.51*	−7.03 ± 2.52*^§^	−19.86 ± 2.56	−11.96 ± 3.53*	−8.24 ± 3.24*^§^	−17.23 ± 2.41	−15.65 ± 3.30	−9.94 ± 3.72*^§^
PSSR	−0.85 ± 0.17	−0.83 ± 0.18	−0.53 ± 0.18*^§^	−1.09 ± 0.22	−0.91 ± 0.43	−0.67 ± 0.42*^§^	−1.04 ± 0.22	−1.11 ± 0.39	−0.73 ± 0.24*^§^
PDSR	1.22 ± 0.29	0.77 ± 0.30*	0.57 ± 0.20*	1.49 ± 0.34	0.90 ± 0.38*	0.72 ± 0.33*	1.31 ± 0.38	0.98 ± 0.57*	0.85 ± 0.38*
**Radial**									
PS	46.08 ± 14.52	32.11 ± 8.12*	20.16 ± 6.89*^§^	35.14 ± 8.69	30.18 ± 8.95	19.11 ± 6.25*^§^	50.49 ± 16.39	44.63 ± 11.19	28.69 ± 11.28*^§^
PSSR	3.02 ± 1.16	1.86 ± 0.88*	0.79 ± 0.55*^§^	2.09 ± 0.97	1.78 ± 1.24*	0.65 ± 0.70*^§^	3.53 ± 1.51	2.97 ± 1.39	1.38 ± 0.89*^§^
PDSR	−4.43 ± 2.06	−1.69 ± 1.34*	−0.84 ± 0.51*^§^	−2.93 ± 1.45	−1.27 ± 1.33*	−0.83 ± 0.4*^s^	−4.21 ± 1.73	−2.55 ± 1.23*	−1.71 ± 0.97*

Notes: The values are the mean ± SD, other abbreviations are the same as in Tables 1 and 2. ^*^ *P* < 0.017 versus normal group; ^§^ *P* < 0.017 versus CA with normal LVEF.

### Association between LV deformation and regional first perfusion

The AL-CA patients with impaired LVEF exhibited a shorter upslope in the basal, mid-ventricular and apical slices (2.52 ± 0.83 vs. 3.59 ± 1.44, 3.01 ± 1.00 vs. 4.09 ± 1.58, and 3.68 ± 1.06 vs. 4.92 ± 1.48, respectively. All *P* < 0.05), reduced MaxSI (30.00 ± 11.95 vs. 38.03 ± 9.68), and longer TTM (52.94 ± 12.30 vs. 41.55 ± 13.24 sec) in the basal segment (result all *P* < 0.05) as compared with the AL-CA patients with normal LVEF. However, no significant differences of MaxSI (40.90 ± 13.33 vs. 44.78 ± 11.84, *P* = 0.083; and 42.90 ± 11.37 vs. 48.30 ± 12.39, *P* = 0.170, respectively) and TTM (39.79 ± 9.42 vs. 37.19 ± 13.80, *P* = 0.470; and 46.07 ± 10.08 vs. 46.68 ± 13.64, *P* = 0.867, respectively) were observed in mid-ventricular and apical slices. As demonstrated in Table 5, the LV longitudinal and circumferential PS and PSSR, and radial PDSR were inversely correlated to upslope and MaxSI in patients with CA. The longitudinal and circumferential PDSR, and radial PS and PSSR were significant and positive associated with perfusion slope and MaxSI.

**Table 5:** Univariable correlations between cardiac deformation parameters and first by-pass parameters in AL-CA subjects with or without LV systolic dysfunction.

Variable	Slope				Time-to-Max signal intensity				Max signal intensity			
	CA with normal LVEF (n = 26)		CA with impaired LVEF (n = 16)		CA with normal LVEF (n = 26)		CA with impaired LVEF (n = 16)		CA with normal LVEF (n = 26)		CA with impaired LVEF (n = 16)	
	R Value	p Value	R Value	p Value	R Value	p Value	R Value	p Value	R Value	p Value	R Value	p Value
**Longitudinal**												
PS	−0.460	<0.0001	−0.557	<0.0001	0.032	0.782	0.227	0.121	−0.361	0.001	−0.375	0.009
PSSR	−0.618	<0.0001	−0.467	0.001	0.477	<0.0001	0.266	0.067	−0.316	0.005	−0.385	0.007
PDSR	0.459	<0.0001	0.528	<0.0001	−0.118	0.302	−0.371	0.009	0.504	<0.0001	0.474	0.001
**Circumferential**												
PS	−0.443	<0.0001	−0.442	0.002	0.072	0.533	0.143	0.333	−0.358	0.001	−0.436	0.002
PSSR	−0.648	<0.0001	−0.463	0.001	0.478	<0.0001	0.250	0.087	−0.405	<0.0001	−0.459	0.001
PDSR	0.389	<0.0001	0.403	0.005	−0.089	0.441	−0.263	0.070	0.443	<0.0001	0.426	0.003
**Radial**												
PS	0.355	0.001	0.360	0.012	−0.040	0.728	−0.078	0.596	0.254	0.025	0.372	0.009
PSSR	0.438	<0.0001	0.370	0.01	−0.343	0.002	−0.141	0.341	0.306	0.006	0.409	0.004
PDSR	−0.357	0.001	−0.375	0.009	−0.003	0.979	0.159	0.280	−0.334	0.003	−0.378	0.008

Note: PS = Peak Strain; PSSR = Peak Systolic Strain Rate; PDR = Peak Diastolic Strain Rate.

## Discussion

Detection and classification of myocardial dysfunction in patients with CA before clinical manifestation of heart failure is essential to predict prognosis^18, 19^ and to determine if CA patients could benefit from autologous stem cell transplantation and high-dose chemotherapy, which is generally accepted as an efficient intervention to improve the survival rates and quality of life in AL amyloidosis^20–22^. Tissue tracking technologies, including speckle tracking echocardiography and feature tracking CMR, have emerged as useful tools for the quantitative measurement of LV subclinical dysfunction prior to reduced LVEF^14^. The echocardiography-derived strains could diagnose and stage myocardial dysfunction in CA patients and these echocardiographic deformation parameters were associated with overall survival in AL amyloidosis patients^6, 19, 23, 24^. However, echocardiographic strain assessment is limited by observer dependency, signal noise, and angle dependency. CMR Feature tracking imaging (FT-CMR) techniques can now assess cardiac deformation without the need for additional CMR scans. The magnetic field strength also had less influence on the intra-observer reproducibility of CMR-derived strain analysis^25^. In previously study, the CMR feature tracking could evaluate almost all myocardial segments by using one single scanning and track all deformation parameters in multi-dimensional directions in normal subjects^26^. However, few studies have evaluated the deformation parameters in AL-CA patients by using CMR feature tracking. Therefore, we investigated the feasibility of 3.0T CMR-based feature tracking algorithm to assess global and regional LV function in patients with AL-CA. By comparison with normal controls, we found CA patients often associated with significantly impaired global and regional longitudinal, circumferential, and radial peak strain as well as peak diastolic strain rate, even in those with preserved LVEF, which is consistent with echocardiographic results^27^, indicated that the CMR-derived strain analysis could detect the early myocardial dysfunction. However, Bhatti *et al*. reported that the preserved global radial strain as well as radial systolic strain rate was detected by CMR in CA patients secondary to multiple myeloma^28^. This discrepancy may partially be explained by the fact that about 36% CA patients with early stage (LV wall thickness less than 1.2 cm) were included in their study while all enrolled patients in our study had an LV wall thickness of more than 1.2 cm. Patients in our study thus may progress to a more advanced disease course even when EF is within normal range, finally resulting in lower global radial strain. Similarly, the typical echocardiographic feature of a baso-apical gradient of longitudinal strain and the relative apical sparing pattern of longitudinal strain in CA patients with normal LVEF is demonstrated in our study by feature tracking CMR^29^. Moreover, CA patients exhibit a significant loss of radial and circumferential strain at the basal slices and relative preservation at the apical level but the absence of basal to apical gradient. Nevertheless, the functionality at apical segments in CA patients with impaired LVEF, often associated with worse clinical status, was compromised, reflected by reduced apical deformations. Our previous study has shown that the LV wall thickness at the basal and mid-cavity segments increased more than at the apex in patients with CA^30^, suggesting relatively greater amyloid deposition in the basal segments and may help explain the underlying pathophysiology for the early dysfunction at the basal segment in CA. In addition, the higher wall stress caused by LV geometry and the largest local radius in basal segments and the greater tendency toward to apoptosis and fibrosis associated with wall stress and turbulent flow in the LV outflow tract in the basal slice may also contribute to the significantly impaired deformation at basal segments in patients with CA^31–33^. As the reduced LV longitudinal strain detected by echocardiography has been considered as an independent predictor of survival in AL amyloidosis and contributed to risk stratification beyond standard clinical and serological parameters in CA^6^, further study is still required to understand whether CMR-derived strain parameters could predict outcome in CA patients.

Given the characteristic CMR finding of subendocardial LGE could be observed in CA with early disease course, as well as the longitudinal myocardial fibers located predominantly in the subendocardium^34^, suggest that subendocardial myocardium is affected first by the disease and the LV myocardial contraction and relaxation were first impaired in the longitudinal direction. In additional, the subendocardium area is most vulnerable to ischemia, since the subendocardial perfusion could be impeded by vascular rarefaction and compression of amyloid deposition. We could presume that coronary microvascular dysfunction may contribute to the impairment of myocardial deformations. Our results indicated that abnormal LV deformation parameters were significantly associated with LV coronary microvascular dysfunction, especially correlated to long-axis function impairment. As the reduced longitudinal strain is associated with worse survival in CA patients, the coronary microvascular dysfunction, which may aggravate the process of deformation impairment, could be the mechanistic link between impaired longitudinal strain and worse outcome in AL patients and thus may help to predict prognosis in CA patients.

Our study had several limitations. First, as the endomyocardial biopsy was not performed for the diagnosis of CA, patients at the early stage of this disease may have been excluded due to the lack of LV hypertrophy exanimated by echocardiography. The myocardial deformations of CA patients at the early stage of disease will be investigated in our future study. Second, the correlation between myocardial deformation abnormalities and the location and quantity of amyloid protein that were related with structural CMR findings such as LGE or T1 mapping and extracellular volume measurements was not carried out in this study. Third, the regional deformation values had a considerably lower intra- and inter-observer reproducibility compared to global values. Although the use of the global strain especially the longitudinal strain has served as a more independent predictor of survival in AL amyloidosis, the global values do not fully reflect the degree of regional wall motion abnormalities. Further development including advanced algorithms and refinement of software may help to resolve this limitation. Finally, the follow up MR scan was not performed. As reported by Hu *et al*., the decrease of longitudinal systolic strain during echocardiographic follow-up examination could predict the risk for imminent death in AL-CA patients^35^. Therefore, whether serial MR scans could provide the same predictive information needs further investigation.

## Conclusion

The 3.0T CMR-derived feature tracking technology could detect impairment of LV myocardial deformation in AL-CA patients, even in CA patients with normal LVEF. In addition, these abnormal myocardial deformation parameters were correlated to impaired LV myocardial microvascular dysfunction. Further study is required to determine if these CMR-derived deformation parameters and coronary microvascular dysfunction may predict prognosis in patients with cardiac amyloidosis.

## Methods

### Study population

This study was approved by the institutional ethics review board of our hospital, and written informed consent was obtained from each participant prior to the investigation. From November 2013 to August 2016, 51 consecutive patients with biopsy-proven AL amyloidosis undergoing CMR were enrolled in the study, according to the following inclusion criteria: (a) AL amyloidosis, initially diagnosed in extra-cardiac tissue using Congo red and immunohistochemical staining and (b) cardiac involvement, confirmed according to echocardiographic criteria^36^. Of 51 participants, 7 patients associated with diabetes (3), hypertension (3), coronary artery disease (4), and severe arrhythmia (2) that may cause myocardium hypertrophy and/or coronary mircovascular dysfunction, and 2 patients whose CMR images were of poor quality were excluded from the study. Consequently, this study involved 42 patients with AL-CA. The mean patient age was 59 years (range 38–81 years). In total, 20 of the 42 patients (47.6%) were females. In our study, patients were diagnosed with CA via typical echocardiographic findings including a mean thickness of the ventricular wall and/or interventricular septum measuring >12 mm in diastole^36^, and an extracardiac tissue biopsy instead of endomyocardial due to its invasive nature and the severity of its potential complications^37^. The extracardiac tissue biopsy was obtained through specimens of kidney (n = 6; 14.3%), bone marrow (n = 36; 85.7%), liver (n = 1; 2.4%), fat (n = 11; 26.2%), rectum (n = 2; 4.8%), skin (n = 6; 14.3%), and tongue (n = 1; 2.4%).

During the same period, 35 normal volunteers (17 males and 18 females; mean age, 50.83 ± 8.67 years; range, 31–64 years) who underwent CMR served as the control group. The exclusion criteria included chronic disease, family history of cardiovascular disease, diabetes, hypertension (>140/90 mmHg), and arrhythmia.

Routine clinical echocardiography was performed for all subjects using two-dimensional transthoracic echocardiography (TEE) with a multiplanar 3.5-MHz probe (IE33; Philips Medical Systems, Andover, MA, USA), according to the guidelines of the American Society of Echocardiography^38^. The interval between the CMR imaging and echocardiography was less than three days.

### CMR imaging

All participants were examined in the supine position using a 3.0-T whole-body scanner (Trio Tim; Siemens Medical Solutions, Erlangen, Germany) following an ECG examination. A dedicated two-element cardiac-phased array coil was used for signal detection. The manufacturer’s standard ECG-triggering device and the breath-hold technique were used to monitor the individuals’ ECG values and breathing, respectively. Following a survey scan, 8–12 continuous CMR cine images were acquired in short-axis slices from the base to the apex using steady-state free-precession (SSFP) sequence (slice thickness 8 mm, repetition time 37.66 ms, echo time 1.2 ms, flip angle 39°, spacing between slices 0 mm). The long-axis two-, three-, and four-chamber view cine series were acquired as well (TR/TE 35.77/1.3 ms, flip angle 40°, field of view 280 mm × 373 mm, matrix size 146 mm × 280 mm, slice thickness 8 mm). For patients with AL-CA, gadobenate dimeglumine (MultiHance 0.5 mmol/ml; Bracco, Milan, Italy) was intravenously injected using an automated injector (Stellant, MEDRAD, Indianola, PA, USA) at a dose of 0.1 ml/kg body weight and a flow rate of 2.5–3.0 ml/s. In addition, a 20-ml saline flush was injected immediately following contrast at a rate of 3.0 ml/s. Perfusion images were acquired in three standard short-axis slices (apical, mid, and basal) and in one slice of the four-chamber view using an inversion-recovery prepared echo-planar imaging sequence (repetition time 210 ms, echo time 1.1 ms, inversion time 90.0 ms, flip angle 10°, field of view 360 mm × 270 mm, acquisition matrix 256 × 192, slice thickness 8 mm). Each set of first-pass perfusion images was completed in 80 cardiac cycles. Late gadolinium enhancement (LGE) imaging was carried out 10–15 min after contrast injection by using an inversion recovery TrueFISP sequence (repetition time/echo time 700/1.31 ms, field of view 320 × 270 mm, and slice thickness 8 mm). All data acquisition was performed during end-inspiratory breath holding.

### CMR data analysis

**Figure 2:**
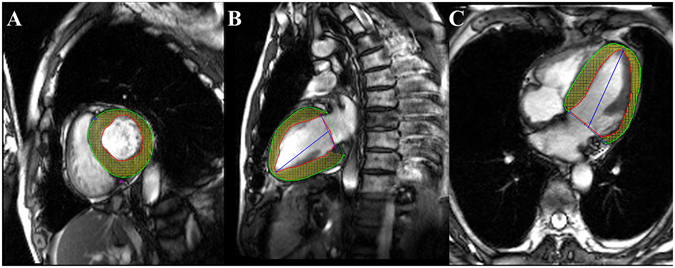
CMR feature tracking in short-axis (**A)**, two-chamber (**B**) and four-chamber long-axis (**C**) cine images at the end-diastole. The red and green curves show the endocardial and epicardial borders, respectively; the yellow dots represent the myocardial voxel points. Abbreviations: CMR, Cardiovascular magnetic resonance.

Image analysis was performed offline by two experienced observers (Li R and Xu HY) using commercial software (cvi^42^; Circle Cardiovascular Imaging, Inc., Calgary, Canada). The endocardial and epicardial traces were performed manually in the serial short-axis slices at the end diastolic and end-systolic phases. Global LV systolic function including LV end-diastolic volume (EDV), end-systolic volume (ESV), and LV ejection fraction (LVEF) were computed. Subsequently, myocardial strain analysis was performed by loaded long-axis 2-chamber, 4-chamber and short-axis slices into the tissue tracking module. In all series, the endocardial and epicardial contours were delineated manually per slice at the end-diastole, and the papillary muscles and moderator bands were carefully excluded **(**Fig. 2). For regional analysis, a 16-segment model (Bull’s eye plot) according to AHA standard segmentation^16^ was constructed on the basis of the analysis of long- and short-axis slices (apical, mid-ventricular, and basal), which included four apical segments (septum, anterior, lateral, and inferior), and six mid-ventricular and basal segments (inferior septum, anterior septum, anterior, anterolateral, inferolateral, and inferior). The global and regional feature tracking parameters were acquired automatically, including myocardial radial, circumferential, and longitudinal peak strain (PS, defined as maximum of the strain in absolute value, over the whole cardiac cycle), peak systolic strain rate (PSSR, defined as maximum of the strain rate in absolute value over all phases starting from diastole till the next systole), and peak diastolic strain rate (PDSR, defined as maximum of the strain rate in absolute value over all phases starting from systole till the next diastole).

**Figure 3:**
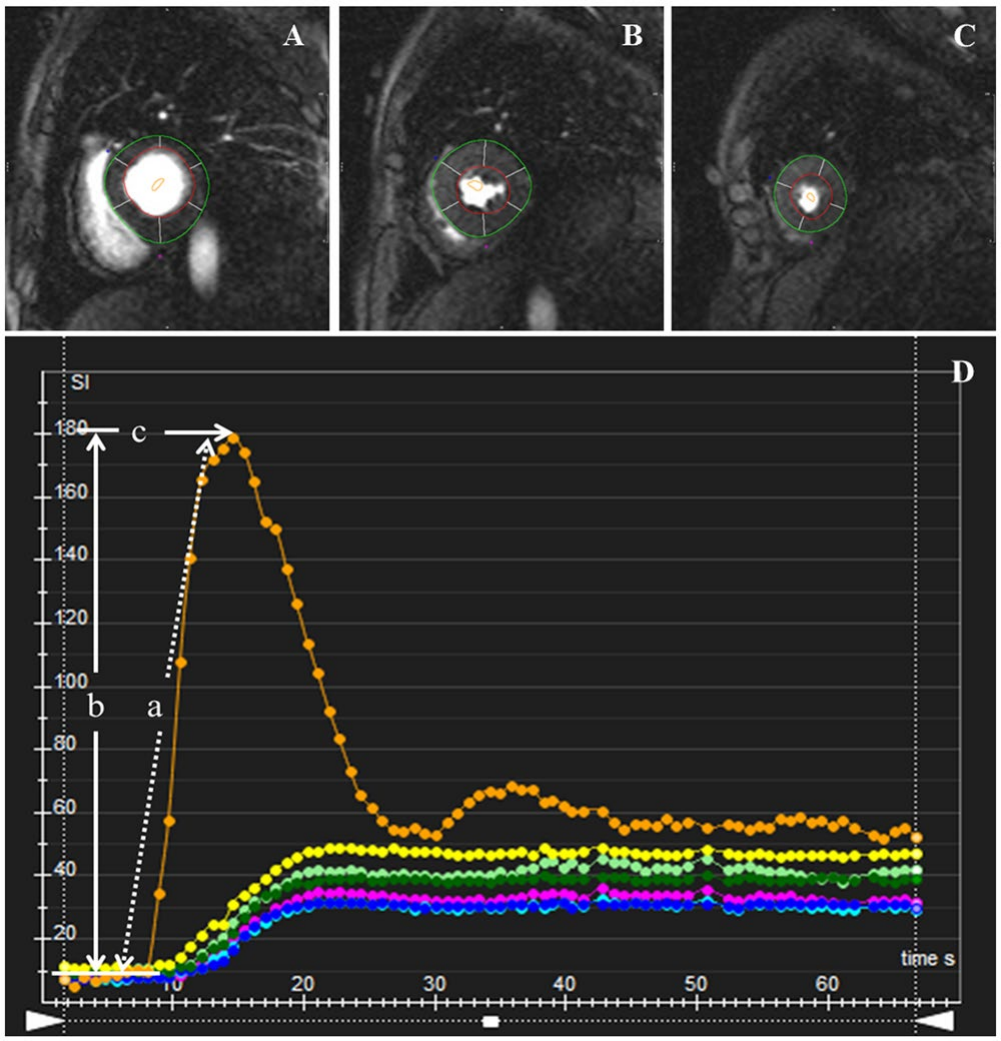
AHA bull’s eye model according to standard segmentation for regional perfusion analysis. Based on this model, segmentation of the LV on myocardial first-pass perfusion images included the following: (**A)** Basal segments; (**B**) Mid-ventricular segments; and (**C**) Apical segments. The first-pass perfusion values including upslope (a), MaxSI (b), and TTM (c) were automatically obtained by signal intensity-time curve derived from myocardial perfusion images (**D**). Abbreviations: MaxSI, Max signal intensity. TTM, time to max signal intensity.

For the evaluation of the LV regional perfusion, the endocardial, epicardial, and blood pool contours of all three sets of first-pass perfusion images (basal, mid-ventricular, and apical) were delineated manually (Fig. 3A–C), and the myocardial signal intensity-time curve was processed. First-pass parameters, including upslope, time to maximum signal intensity (TTM), and max signal intensity (MaxSI), were consequently obtained from the myocardial signal intensity–time curve (Fig. 3D). The diagnosis of LGE was obtained by visual assessment, required viewing an elevated signal in the myocardium within two orthogonal views; the main LGE pattern for each patient was recorded.

### Reproducibility

Intra-observer variability of myocardial deformation parameters was obtained by comparison of measurements by the same observer (Li R and Xu HY) in 20 random cases. The measuring time interval of the same observer is 2 weeks. Inter-observer variability was accessed by another two independent double-blinded observers (Liu X and Shi K).

### Statistics Analysis

All statistical analyses were performed using SPSS software (Version 19.0 for Windows; SPSS, Chicago, IL, USA). The results are expressed as the mean ± standard deviation (SD). The Shapiro-Wilk test was performed to determine normality. Inter- and intra-observer variability was assessed using the coefficient of variation (CV), and the comparison between global and regional CV was performed by Mann–Whitney *U* test. A one-way ANOVA was used to evaluate LV function of the normal and AL-CA groups. A Mann–Whitney *U* test was used to compare regional myocardial perfusion between AL-CA groups, and to compare the LV myocardial deformation parameters among the normal subjects, the AL-CA patient with preserved LVEF and AL-CA patients with impaired LVEF using Bonferroni’s correction for multi-group comparisons. Spearman’s rank correlation analysis was performed to examine the correlation between myocardial deformation parameters and regional myocardial perfusion. A P-value < 0.05 was considered to indicate a statistically significant difference.

### Ethical Approval and Consent to participate

The local ethics committee (Medical Ethics Committee of Sichuan University) approved this retrospective study. The institutional review board approved the study protocol and the requirement for informed consent was waived off.

## References

[1] Falk RH (2005). Diagnosis and management of the cardiac amyloidoses. Circulation.

[2] Kristen AV (2007). Non-invasive predictors of survival in cardiac amyloidosis. Eur J Heart Fail.

[3] Gertz MA (2011). Immunoglobulin light chain amyloidosis: 2011 update on diagnosis, risk-stratification, and management. Am J Hematol.

[4] Selvanayagam JB, Hawkins PN, Paul B, Myerson SG, Neubauer S (2007). Evaluation and management of the cardiac amyloidosis. J Am Coll Cardiol.

[5] Lee GY (2015). Visual Assessment of Relative Apical Sparing Pattern Is More Useful Than Quantitative Assessment for Diagnosing Cardiac Amyloidosis in Borderline or Mildly Increased Left Ventricular Wall Thickness. Circ J.

[6] Liu D (2013). Impact of regional left ventricular function on outcome for patients with AL amyloidosis. PLoS One.

[7] Hor KN (2010). Comparison of magnetic resonance feature tracking for strain calculation with harmonic phase imaging analysis. JACC Cardiovasc Imaging.

[8] Schuster A, Hor KN, Kowallick JT, Beerbaum P, Kutty S (2016). Cardiovascular Magnetic Resonance Myocardial Feature Tracking: Concepts and Clinical Applications. Circ Cardiovasc Imaging.

[9] Kerwin WS, Prince JL (1998). Cardiac material markers from tagged MR images. Med Image Anal.

[10] Chuang ML (2000). Importance of imaging method over imaging modality in noninvasive determination of left ventricular volumes and ejection fraction: Assessment by two- and three-dimensional echocardiography and magnetic resonance imaging. J Am Coll Cardiol.

[11] Claus P, Omar AM, Pedrizzetti G, Sengupta PP, Nagel E (2015). Tissue Tracking Technology for Assessing Cardiac Mechanics: Principles, Normal Values, and Clinical Applications. JACC Cardiovasc Imaging.

[12] Augustine D (2013). Global and regional left ventricular myocardial deformation measures by magnetic resonance feature tracking in healthy volunteers: comparison with tagging and relevance of gender. J Cardiovasc Magn Reson.

[13] Pedrizzetti G, Claus P, Kilner PJ, Nagel E (2016). Principles of cardiovascular magnetic resonance feature tracking and echocardiographic speckle tracking for informed clinical use. J Cardiovasc Magn Reson.

[14] Smiseth OA, Torp H, Opdahl A, Haugaa KH, Urheim S (2016). Myocardial strain imaging: how useful is it in clinical decision making?. Eur Heart J.

[15] Hor KN (2009). Circumferential strain analysis identifies strata of cardiomyopathy in Duchenne muscular dystrophy: a cardiac magnetic resonance tagging study. J Am Coll Cardiol.

[16] Rosen BD (2006). Hypertension and smoking are associated with reduced regional left ventricular function in asymptomatic: individuals the Multi-Ethnic Study of Atherosclerosis. J Am Coll Cardiol.

[17] Dorbala S (2014). Coronary microvascular dysfunction is related to abnormalities in myocardial structure and function in cardiac amyloidosis. JACC Heart Fail.

[18] Koyama J, Falk RH (2010). Prognostic significance of strain Doppler imaging in light-chain amyloidosis. JACC Cardiovasc Imaging.

[19] Buss SJ (2012). Longitudinal left ventricular function for prediction of survival in systemic light-chain amyloidosis: incremental value compared with clinical and biochemical markers. J Am Coll Cardiol.

[20] Perz JB (2004). High-dose melphalan with autologous stem cell transplantation after VAD induction chemotherapy for treatment of amyloid light chain amyloidosis: a single centre prospective phase II study. Br J Haematol.

[21] Skinner M (2004). High-dose melphalan and autologous stem-cell transplantation in patients with AL amyloidosis: an 8-year study. Ann Intern Med.

[22] Cibeira MT (2011). Outcome of AL amyloidosis after high-dose melphalan and autologous stem cell transplantation: long-term results in a series of 421 patients. Blood.

[23] Baccouche H (2012). Differentiating cardiac amyloidosis and hypertrophic cardiomyopathy by use of three-dimensional speckle tracking echocardiography. Echocardiography.

[24] Liu D (2014). Predictive value of assessing diastolic strain rate on survival in cardiac amyloidosis patients with preserved ejection fraction. PLoS One.

[25] Schuster A (2013). The intra-observer reproducibility of cardiovascular magnetic resonance myocardial feature tracking strain assessment is independent of field strength. Eur J Radiol.

[26] Andre F (2015). Age- and gender-related normal left ventricular deformation assessed by cardiovascular magnetic resonance feature tracking. J Cardiovasc Magn Reson.

[27] Sun JP (2009). Differentiation of hypertrophic cardiomyopathy and cardiac amyloidosis from other causes of ventricular wall thickening by two-dimensional strain imaging echocardiography. Am J Cardiol.

[28] Bhatti, S. *et al*. Myocardial strain pattern in patients with cardiac amyloidosis secondary to multiple myeloma: a cardiac MRI feature tracking study. *Int J Cardiovasc Imaging* [Epub ahead of print], doi:10.1007/s10554-016-0998-6 (2016). 10.1007/s10554-016-0998-6 27743139

[29] Phelan D (2012). Relative apical sparing of longitudinal strain using two-dimensional speckle-tracking echocardiography is both sensitive and specific for the diagnosis of cardiac amyloidosis. Heart.

[30] Li R (2016). Regional myocardial microvascular dysfunction in cardiac amyloid light-chain amyloidosis: assessment with 3T cardiovascular magnetic resonance. J Cardiovasc Magn Reson.

[31] Jiang L, Huang Y, Hunyor S, dos Remedios CG (2003). Cardiomyocyte apoptosis is associated with increased wall stress in chronic failing left ventricle. Eur Heart J.

[32] Balzer P (1999). Regional assessment of wall curvature and wall stress in left ventricle with magnetic resonance imaging. Am J Physiol.

[33] Grossman W, Jones D, McLaurin LP (1975). Wall stress and patterns of hypertrophy in the human left ventricle. J Clin Invest.

[34] Kocica MJ (2006). The helical ventricular myocardial band: global, three-dimensional, functional architecture of the ventricular myocardium. Eur J Cardiothorac Surg.

[35] Hu K (2015). Impact of monitoring longitudinal systolic strain changes during serial echocardiography on outcome in patients with AL amyloidosis. Int J Cardiovasc Imaging.

[36] Gertz MA (2005). Definition of organ involvement and treatment response in immunoglobulin light chain amyloidosis (AL): a consensus opinion from the 10th International Symposium on Amyloid and Amyloidosis, Tours, France, 18–22 April 2004. Am J Hematol.

[37] Pellikka PA (1988). Endomyocardial biopsy in 30 patients with primary amyloidosis and suspected cardiac involvement. Arch Intern Med.

[38] Cerqueira MD (2002). Standardized myocardial segmentation and nomenclature for tomographic imaging of the heart. A statement for healthcare professionals from the Cardiac Imaging Committee of the Council on Clinical Cardiology of the American Heart Association. Circulation.

